# Supplementary material for: Association Between the Activity Space Exposure to Parks in Childhood and Adolescence and Cognitive Aging in Later Life
Source: Int J Environ Res Public Health. 2019 Feb 21;16(4):632. doi: 10.3390/ijerph16040632 (PMC6406333; doi:10.3390/ijerph16040632)
Supplement: Supplementary file 1 [file ijerph-16-00632-s001.pdf]

Supplementary Materials

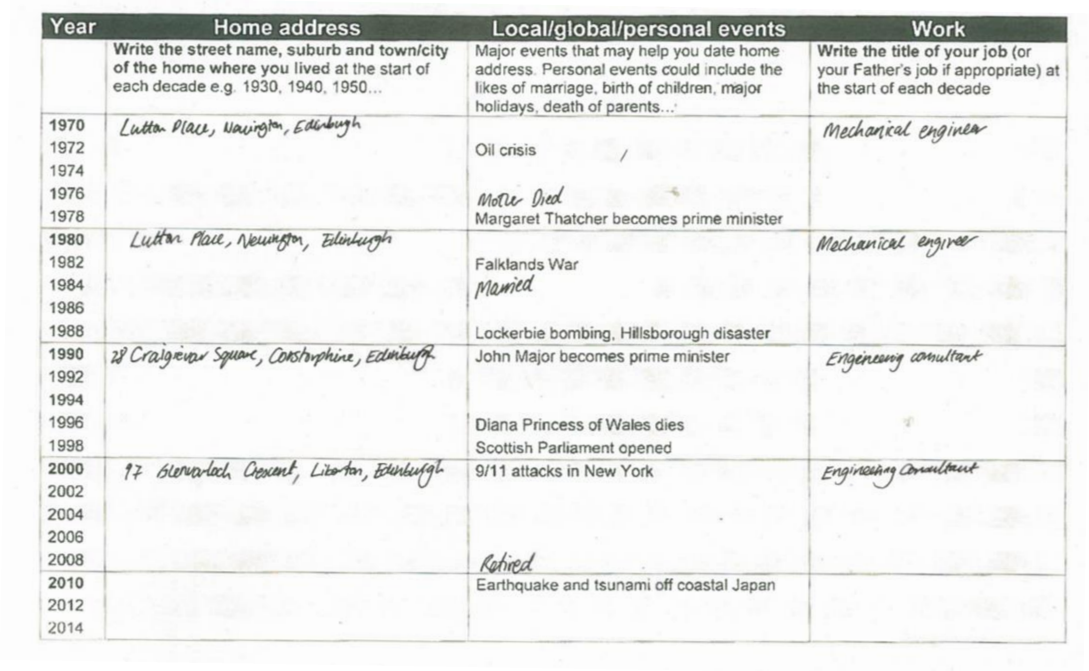

Year	Home address	Local/global/personal events	Work
	Write the street name, suburb and town/city of the home where you lived at the start of each decade e.g. 1930, 1940, 1950…	Major events that may help you date home address. Personal events could include the likes of marriage, birth of children, major holidays, death of parents…	Write the title of your job (or your Father's job if appropriate) at the start of each decade
1970	Lutton Place, Newington, Edinburgh		Mechanical engineer
1972		Oil crisis	
1974			
1976		Mother Died	
1978		Margaret Thatcher becomes prime minister	
1980	Lutton Place, Newington, Edinburgh		Mechanical engineer
1982		Falklands War	
1984		Married	
1986			
1988		Lockerbie bombing, Hillsborough disaster	
1990	28 Craigievar Square, Corstorphine, Edinburgh	John Major becomes prime minister	Engineering consultant
1992			
1994			
1996		Diana Princess of Wales dies	
1998		Scottish Parliament opened	
2000	17 Glenvarloch Crescent, Liberton, Edinburgh	9/11 attacks in New York	Engineering Consultant
2002			
2004			
2006			
2008		Retired	
2010		Earthquake and tsunami off coastal Japan	
2012			
2014			

Figure S1. Example of Lifegrid questionnaire. n.b. This is fictional data to preserve anonymity of the participants.

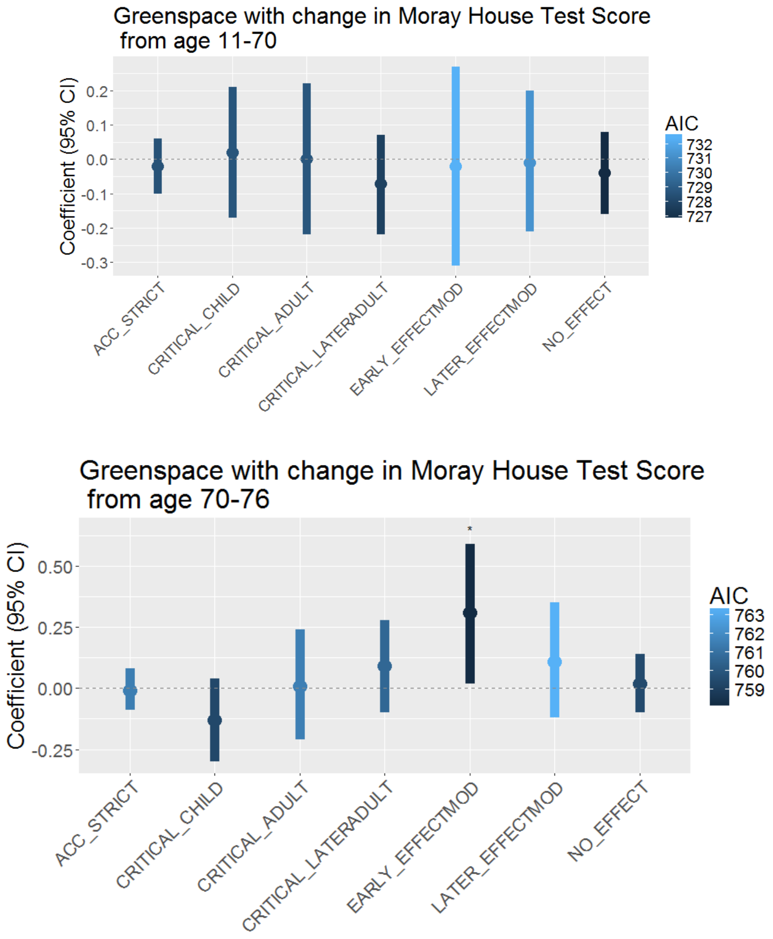

Figure S2. Life-course model selection.

Table S1. Comparison between LBC1936 analysis sample and residential life course sample.

Characteristic	LBC1936 analysis sample (n=281)	LBC1936 residential life course sample (n=592)
	Mean (±SD); N (%)	Mean (±SD); N (%)
Sex		
Female	134 (48)	279 (47)
Father's Occupational Social class		
Professional-managerial (I/II)	62 (22)	153 (26)
Skilled, partly skilled, unskilled (III/IV/V)	203 (72)	403 (68)
NA	16 (6)	36 (6)
Participant's Occupational Social class		
Professional-managerial (I/II)	151 (54)	354 (60)
Skilled, partly skilled, unskilled (III/IV/V)	127 (45)	231 (39)
NA	3 (1)	7 (1)
Educational attainment		
Degree	31 (11)	105 (18)
Semi-professional/professional qualifications	30 (11)	78 (13)
A-level or equivalent	45 (16)	97 (16)
O-level of equivalent	132 (47)	223 (37)
No qualification	42 (15)	88 (15)
NA	1 (0)	1 (0)
MHT change 70-76	0.01 ± 0.95	0.06 ± 0.93
NA	0 (0)	27 (5)

Table S2. Relationship between childhood and adolescent activity space park availability and cognitive change in later life.

Public Parks Availability [a]	Change in cognitive function from age 70 to age 76 on Moray House Test
Large buffers	
Childhood Activity Space * Adulthood Home	0.17 (-0.11 - 0.44) [0.2306]
Adolescent Activity Space * Adulthood Home	0.16 (-0.08 -0.41) [0.1978]
Moderate buffers	
Childhood Activity Space * Adulthood Home	0.22 (-0.07 - 0.51) [0.1475]
Adolescent Activity Space * Adulthood Home	0.27 (0.00 - 0.55) [0.0496]
Small buffers	
Childhood Activity Space * Adulthood Home	0.24 (-0.07 - 0.54) [0.1270]
Adolescent Activity Space * Adulthood Home	0.14 (-0.16 -0.44) [0.3597]

[a] Large buffers equates to buffer sizes of 1,500 m surrounding the home, 1,500 m surrounding the school and 300 m surrounding the route to school; moderate buffers equates to buffer sizes of 1,000 m surrounding the home, 1,000 m surrounding the school and 100 m surrounding the route to school; small buffers equates to buffer sizes of 500 m surrounding the home, 500 m surrounding the school and 100 m surrounding the route to school. Adulthood Home corresponds to the size of the buffer for the childhood home, i.e. for small buffers adulthood home is buffer size of 500 m. [a] Park availability determined as % of area within 1,000 m buffer surrounding home and school, and 200m buffer surrounding route to school [b] OR (95%CI) [p-value] [c] Adjusted for sex, father's occupational social class, people per room in childhood home, and childhood smoking, adulthood OSC, alcohol consumption and smoking status * Interaction term.
